# CRISPR-M: Predicting sgRNA off-target effect using a multi-view deep learning network

**DOI:** 10.1371/journal.pcbi.1011972

**Published:** 2024-03-14

**Authors:** Jialiang Sun, Jun Guo, Jian Liu

**Affiliations:** 1 College of Computer Science, Nankai University, Tianjin, China; 2 College of Software, Northeastern University, Shenyang, China; 3 Centre for Bioinformatics and Intelligent Medicine, Nankai University, Tianjin, China; University of Illinois at Urbana-Champaign, UNITED STATES

## Abstract

Using the CRISPR-Cas9 system to perform base substitutions at the target site is a typical technique for genome editing with the potential for applications in gene therapy and agricultural productivity. When the CRISPR-Cas9 system uses guide RNA to direct the Cas9 endonuclease to the target site, it may misdirect it to a potential off-target site, resulting in an unintended genome editing. Although several computational methods have been proposed to predict off-target effects, there is still room for improvement in the off-target effect prediction capability. In this paper, we present an effective approach called CRISPR-M with a new encoding scheme and a novel multi-view deep learning model to predict the sgRNA off-target effects for target sites containing indels and mismatches. CRISPR-M takes advantage of convolutional neural networks and bidirectional long short-term memory recurrent neural networks to construct a three-branch network towards multi-views. Compared with existing methods, CRISPR-M demonstrates significant performance advantages running on real-world datasets. Furthermore, experimental analysis of CRISPR-M under multiple metrics reveals its capability to extract features and validates its superiority on sgRNA off-target effect predictions.

## Author summary

Genome editing using the CRISPR-Cas9 system, particularly base substitutions directed by guide RNA, holds immense potential for applications in gene therapy and agricultural productivity. However, the risk of unintended off-target effects poses a challenge, as misdirection of the Cas9 endonuclease can lead to unintended genome alterations. While computational methods exist for predicting off-target effects, there remains a need for encoding methods with more representation space and deep learning models with generalization capability and the adaptability. This paper introduces CRISPR-M, an innovative approach addressing the limitations of existing methods in predicting off-target effects, especially for target sites with indels and mismatches. CRISPR-M employs a novel encoding scheme and a multi-view deep learning model, combining convolutional neural networks and bidirectional long short-term memory recurrent neural networks. The three-branch network structure enhances the prediction accuracy by considering multiple perspectives. Compared with previous representative methods, CRISPR-M exhibits remarkable performance advantages when applied to real-world datasets. The experimental evaluation of CRISPR-M, assessed by various metrics such as ROC, PRC, GC content and melting temperature, demonstrates its ability to extract meaningful features and establishes its superiority in predicting off-target effects of sgRNA.

## Introduction

The Clustered Regularly Interspaced Short Palindromic Repeats (CRISPR) associated with protein Cas9 (Cas9) system (CRISPR-Cas9 system) is an advanced technology that can be applied in genome engineering [1–4]. It is a two-component system, in which the first component Cas9 endonuclease is guided to the DNA target sequence upstream of PAM (protospacer adjacent motif) and complementary to the second component sgRNA (single-guide RNA), allowing the bases of the target sequence to be edited [5,6]. It has the potential to be applied in gene therapy and agriculture productivity [7–9]. The sgRNA represents a synthetic adaptation of the native two-piece guide RNA complex, combining the crRNA for directing Cas9 to the target site and the trans-activating crRNA (tracrRNA) acting as a binding scaffold, thereby streamlining the CRISPR-Cas9 system for precise and efficient genome editing[6]. The CRISPR-Cas9 system still needs to be further optimized, as its off-target effect could diminish the specificity of gene editing [10,11]. Quantifying off-target effects by using sequencing technologies, such as GUIDE-seq (genome-wide, unbiased identification of DSBs enabled by sequencing) [12], SITE-seq (selective enrichment and identification of tagged genomic DNA ends by sequencing) [13], CIRCLE-seq (circularization for in vitro reporting of cleavage effects by sequencing) [14] and Digenome-seq (in vitro Cas9-digested whole-genome sequencing) [15], could contribute to the optimization of CRISPR-Cas9 systems. Introducing in silico methods to quantify off-target effects is promising in saving time, money and labor cost [16]. Furthermore, using machine learning techniques to capture latent features is promising and helpful to enhance the efficiency and specificity of CRISPR-Cas9 systems [17].

Early hypothesis-driven in silico tools such as MIT [11], CCTop [18] and CROP-IT [19] are centered on empirically determined hand-crafted rules including sensitivity of number, position and distribution of mismatch sites [11], distance to the closest annotated exon [18] and chromatin state information [19]. Based on the rule set regarding mismatch substitution types and mismatch positions, CFD [20,21] performs predictions of sgRNA off-target effects and outperforms previous hypothesis-driven methods (i.e., CCTop score, CROP-IT score and MIT score) [22]. Based on a two-layer machine learning model, ELEVATION [23] uses features derived from the mismatch sites between intended target sequences and potential off-target sequences to implement off-target prediction. These previous studies have encountered two main difficulties: i) hand-crafted features may increase specialization and heterogeneity, resulting in weak generalization ability of machine learning models [20], ii) The machine learning approaches above are limited in the ability of mining data features and making predictions.

Using pairs of on-target and off-target sequences encoded by ONE-HOT encoding and OR operations as input features, CNN_std [24] uses the convolutional neural networks to perform off-target predictions. Extracting features from sgRNA and DNA sequence pairs, AttnToMismatch_CNN [25] constructs a model based on a self-attention-based transformer architecture [26] combined with CNN to deal with off-target predictions. Using on-target sequences, off-target sequences and epigenetic features as training features, DeepCRISPR [27] constructs a convolutional neural network model to achieve off-target predictions. However, these methods consider mismatches in the off-target prediction only, and ignore insertions/deletions (i.e., indels) between target DNA and guide RNA sequences [28]. Recent approaches incorporate insertions and deletions into training features, for example, CRISPR-Net [29] trains a deep learning model based on the Inception architecture and the LSTM (Long Short-Term Memory) architecture using seven-bit encoded features. R-CRISPR [30] uses an encoding approach similar to CRISPR-Net, using the RepVGG architecture to enhance the deep learning model. CRISPR-IP [31] compresses the encoding scheme and adds an attention layer to the deep learning model. However, these existing deep learning approaches depend on OR operations to artificially compress the encoding scheme for on-target and off-target sequences, which limits the representation space of the input features to some extent. Meanwhile, they use models with relatively few layers, which limit the generalization capability and the adaptability of processing datasets with multiple different characteristics.

To deal with the issues above and enhance off-target effect prediction, in this paper, we propose a novel multi-view deep learning model with a new feature encoding scheme, named CRISPR-M, regarding sgRNA off-target effect prediction for target sites containing indels and mismatches. In particular, we firstly design three views encoding pairs of on- and off-target sequence, on-target sequences and off-target sequences, aiming to capture the features of associations between on- and off-target sequences, the features of on-target sequences and the features of off-target sequences, respectively. Secondly, we develop a dictionary of base pairs and individual bases to encode the features of multi-views above, with the assistance of word embedding and positional encoding. Based on the convolutional neural network and the recurrent neural network, we propose a multi-branch deep learning model called CRISPR-M, associated with these three input features. Experimental evaluation on real-world datasets demonstrates that CRISPR-M outperforms previous approaches in terms of ROC (receiver operating characteristic curve), PRC (precision recall curve), Spearman correlation rank coefficient and F-score. In addition, experimental results on encoding scheme, epigenetic features and sampling scheme also validate the superiority of our proposed approach on sgRNA off-target effect predictions. Finally, we perform a visual analysis of the features captured by CRISPR-M and reveal the influence of mismatches and indels on off-target effects.

## Results

### Datasets

We collect two categories of datasets for model learning and validation. One category contains mismatches and indels, i.e., datasets CIRCLE and GUIDE_I in Table 1, and the other category contains mismatches only, i.e., other datasets in Table 1. The CIRCLE dataset identifies 340 active off-target loci samples containing indels and 7031 active off-target loci samples containing mismatch only using the CIRCLE-seq technique. The Cas-OFFinder tool [32] is used to search the genomes and obtain 252,539 inactive off-target loci samples containing indel and 325,039 inactive off-target loci samples containing mismatch only. Note that the CIRCLE dataset is derived from the experimental data of 10 gRNAs and contains sufficient off-target samples for each gRNA, which is suitable for ten-fold cross validation. The GUIDE_I dataset also contains indel samples, but contains only 60 active off-target loci samples. For the rest datasets in Table 1, we use PKD (Protein knockout detection), SITE, GUIDE_II, and GUIDE_III for the mismatch-only experiments, and HEK293T and K562 for the experiments regarding epigenetic features. PKD has sufficient data for active off-target sites, but insufficient data for inactivated off-target sites. SITE has sufficient active off-target sites and inactivated off-target sites. GUIDE_II and GUIDE_III have sufficient data for inactive off-target loci samples, but only a small number of active off-target loci samples.

**Table 1:** Datasets used for model learning and validation.

Dataset aliases	Total Sites	Off-Target Sites	Indel	gRNAs
CIRCLE [14]	584949	7371	430	10
GUIDE_I [23]	213943	60	13	6
Protein knockout detection (PKD) [20]	4853	2273	N/A	65
SITE [13]	217733	3767	N/A	9
GUIDE_II [33]	95829	54	N/A	5
GUIDE_III [23]	383463	56	N/A	22
HEK293T [27]	132914	536	N/A	18
K562 [27]	20319	120	N/A	12

### Performance measures

In the Results section, we use a series of metrics to evaluate the performance of our proposed approaches. In particular, Accuracy, Precision, Recall, F1 Score, F2 Score, AUROC (Area Under the Receiver Operating Characteristic), AUPRC (Area Under the Precision-Recall Curve) and Spearman rank correlation coefficient (SRCC) are used for comparisons. The detailed metrics are as follows:

$$Accuracy=\frac{TP+TN}{TP+FP+FN+TN}$$ (1)

$$Precision=\frac{TP}{TP+FP}$$ (2)

$$Recall=\frac{TP}{TP+FN}$$ (3)

$$F_{1}=\frac{2\times Precision\times Recall}{Precision+Recall}$$ (4)

$$F_{2}=\frac{5\times Precision\times Recall}{4\times Precision+Recall}$$ (5)

$$SRCC=1-\frac{6\times \sum_{i=1}^{N}{\left|R(X_{i})-R(Y_{i})\right|}^{2}}{N\times (N^{2}-1)}$$ (6)

*TP* denotes the number of true positive examples, *FP* denotes the number of false positive examples, *FN* denotes the number of false negative examples and *TN* denotes the number of true negative examples. *N* denotes the number of test samples. R(*X*) and R(*Y*) are the ranking of the two sets of variables *X* and *Y*, representing the predicted values and real values. We use the Spearman rank correlation coefficient to measure the correlation between the predicted values and real values. We choose the F1 Score as the metric based on a combination of precision and recall. We also used the F2 Score with increased the weighting of Recall as another evaluation metric. For ROC and PRC, we choose the macro-averaging calculation method to minimize the effect of the different number of datasets. In addition, the GC content of sgRNA and the melting temperature [34,35] between sgRNA and off-target site sequence are used for visual analysis.


$$GC\;content=\frac{N_{G}+N_{C}}{sgRNA\;length}$$ (7)

$$T_{m}=\frac{\Delta H{}^{\circ}}{R\;\ln\frac{C_{t}}{n}+\Delta S{}^{\circ}}$$ (8)

Here *N*_*G*_ indicates the amount of guanine in the sgRNA, and *N*_*C*_ indicates the amount of cytosine in the sgRNA. T_m_ indicates the melting temperature. *R* indicates the gas constant. *ΔH°* and *ΔS°* indicate calculated enthalpy and entropy changes. *C*_*t*_ indicates the total strand concentration. The *n* indicates the symmetry factor, which is 1 for self-complementary strands and 4 for non-self-complementary strands.

### Comparisons on the target sites containing both mismatches and indels

Two datasets CIRCLE [14] and GUIDE_I [23] containing mismatches and indels are used in the experiments. To verify the sgRNA off-target effect prediction capability of CRISPR-M, we split the CIRCLE dataset into ten parts by the corresponding sgRNA of the samples for leave-one-gRNA-out cross-validation (LOGOCV), at first. Then, we use the CIRCLE dataset as the training set and the GUIDE_I dataset as the validation set (depicted as CIRCLE_GUIDE) to perform the comparisons. Note that GUIDE_I dataset is not used as the training set due to the relatively small amount of active off-target data, which could lead to unstable training of the model. We compare CRISPR-M with previous representative approaches CRISRP-IP [31], R-CRISPR [30] and CRISPR-Net [29]. In addition, we retrain the competition models on the same dataset to ensure fairness of the comparisons.

**Fig 1:**
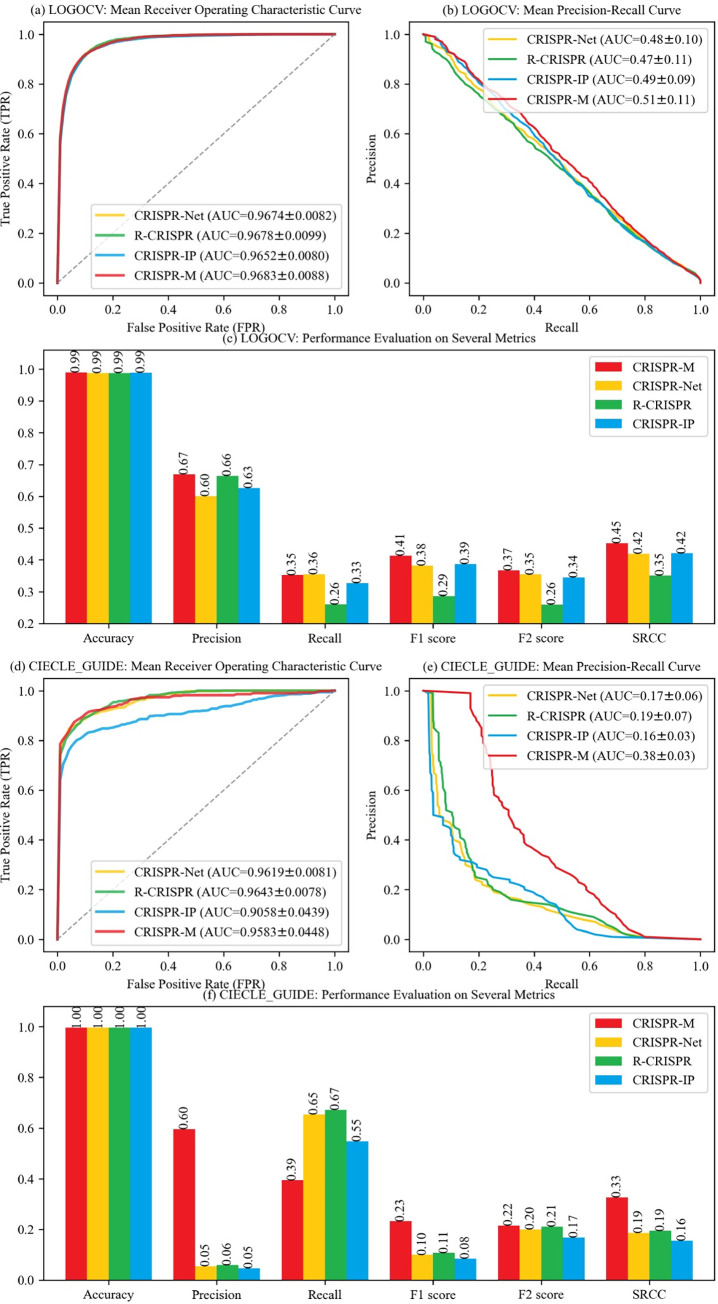
Comparisons on-target sites containing both mismatches and indels.

Fig 1A–1C show the experimental results of the LOGOCV on dataset CIRCLE. CRISPR-M performs the best on both the ROC and the PRC (the area under ROC, i.e., AUROC, is about 0.9683, and the area under PRC, i.e., AUPRC is about 0.51). In particular, Fig 1(A) shows that CRISPR-M performs the best on ROC, and Fig 1(B) shows that CRISPR-M outperforms CRISRP-IP, R-CRISPR and CRISPR-Net on PRC (in excess of 2%-4%). R-CRISPR outperforms CRISRP-IP and CRISPR-Net in terms of ROC (AUROC≈0.9678). CRISPR-IP outperforms R-CRISPR and CRISPR-Net in terms of PRC (AUPRC≈0.49). In Fig 1(C), for CRISPR-M, CRISRP-IP, R-CRISPR and CRISPR-Net, similar results on Precision and Recall are obtained. In addition, we see that, CRISPR-M outperforms CRISRP-IP, R-CRISPR and CRISPR-Net in terms of F1 Score and F2 Score. Further, CRISPR-M outperforms the other three models in terms of the Spearman rank correlation coefficient, which is used to reveal the correlation between the predicted values and real values. Fig 1D–1F show the experimental results on dataset CIRCLE_GUIDE. In Fig 1(D), we see that CRISPR-IP performs the worst in terms of ROC, and similar results are obtained by using CRISRP-M, R-CRISPR and CRISPR-Net. In Fig 1(E), we see that CRISRP-M performs the best in terms of PRC, and show twofold increases in PRC compared with CRISRP-IP, R-CRISPR and CRISPR-Net. The difference between the AUROC and the AUPRC results is due to the class imbalance within the CRISPR experimental datasets. Successful gene edits (positive instances) are rare events, in contrast to the vast number of non-edited instances (negative instances). The AUROC metric is less sensitive to imbalanced class distributions, and its calculation relies on the False Positive Rate (FPR), which may be influenced by the overwhelming number of negative instances. In contrast, the AUPRC places a stronger emphasis on the precision-recall trade-off, making it more suitable for evaluating performance in scenarios of imbalanced class proportions. In other words, CRISRP-M shows better results in terms of AUPRC, suggesting its effectiveness in correctly classifying positive instances, which is important in applications of accurately identifying the minority class. In Fig 1(F), we see that CRISRP-M performs the best in terms of Precision, F1 Score, F2 Score and SRCC, compared with CRISRP-IP, R-CRISPR and CRISPR-Net.

Overall, in terms of Precision and Recall, these four approaches have similar results of the LOGOCV on dataset CIRCLE, but CRISPR-IP, R-CRISPR and CRISPR-Net show huge difference and unbalance of Precision and Recall on dataset CIRCLE_GUIDE. Because the LOGOCV experiment uses a single CIRCLE dataset for training and validating, and CIRCLE_GUIDE consists of datasets from different sources (it takes CIRCLE and GUIDE_I as the training set and validating set respectively) and GUIDE_I has more unbalanced ratio of positive and negative examples, making the validation challenging compared with the LOGOCV on CIRCLE. In addition, CRISPR-M performs best on the metrics of PRC, SRCC, F1 Score and F2 Score. These results further demonstrate that CRISPR-M has better generalization capability for sgRNA off-target effect predictions on-target sites containing mismatches and indels.

### Comparisons on mismatches-only sgRNA-target prediction

In this section, we test CRISPR-M on datasets containing mismatches-only samples. Four datasets, SITE [13], PKD (Protein knockout detection) [20], GUIDE_II [33] and GUIDE_III [23], are used in the experiments. These four datasets are divided into two groups for 2-fold cross-validation, one group consisting of the SITE dataset and the other group consisting of the rest datasets. Three representative methods CRISPR-Net, R-CRISPR and CRISPR-IP for handling both mismatches and indels are compared with CRISPR-M. Although these three methods have proven their superiority over earlier methods, three representative approaches CFDScoring [20], CNN_std [24] and DeepCRISPR [27] for handling mismatches only are also compared with CRISPR-M for a more general comparison.

**Fig 2:**
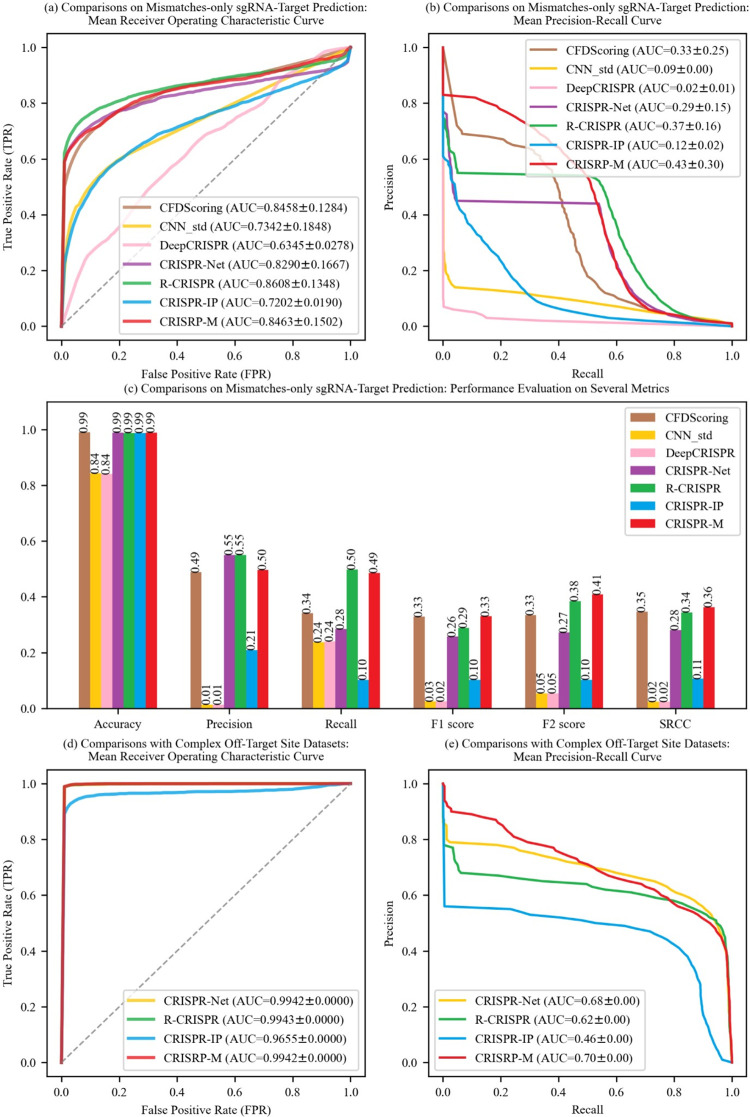
Comparisons on mismatches-only sgRNA-target prediction and complex off-target site datasets.

Fig 2(A) shows the ROC results of these seven approaches. CRISPR-M (AUROC≈0.8463) has the second highest AUROC, about 1.5% lower than R-CRISPR. As shown in Fig 2(B), CRISPR-M (AUPRC≈0.43) outperforms the other six approaches in terms of PRC (in excess of 6% at least). In Fig 2(C), we see that DeepCRISPR performs the worst in terms of Accuracy, Precision, Recall, F1 Score, F2 Score and SRCC. This may be due to the fact that DeepCRISPR is designed for epigenetic features and is not suitable for the tests on base-sequence-only features. Compared with these six approaches, CRISPR-M achieves optimal performance on Accuracy, F1 Score, F2 Score and SRCC. These results further demonstrate that CRISPR-M is not only good at processing indels, but also has excellent prediction capability on mismatch-only samples.

### Comparisons with complex off-target site datasets

To further validate the performance of CRISPR-M, we integrate datasets CIRCLE [14], GUIDE_I [23], SITE [13], PKD [20], GUIDE_II [32] and GUIDE_III [23] with different characteristics for these experiments. We merge CIRCLE and SITE as the training set, and integrate GUIDE_I, GUIDE_II, GUIDE_III and PKD as the validation set.

As shown in Fig 2(D), the AUROCs of CRISRP-M, R-CRISPR and CRISPR-Net are approximately equal to 0.994, except for CRISPR-IP which has the lowest AUROC (approximately 0.9655). In Fig 2(E), we see that CRISPR-IP (AUPRC = 0.46) performs the worst and CRISPR-M (AUPRC = 0.70) performs the best. This is consistent with the result shown in Fig 2(B), where the AUPRCs of CRISPR-IP and CRISPR-M is 0.12 and 0.43, respectively. The poor performance of CRISPR-IP may be due to the excessive compressive encoding. Overall, CRISPR-M outperforms the other approaches, still performs robustly, and shows better adaptability in more complex off-target site datasets.

### Comparisons of encoding schemes

**Fig 3:**
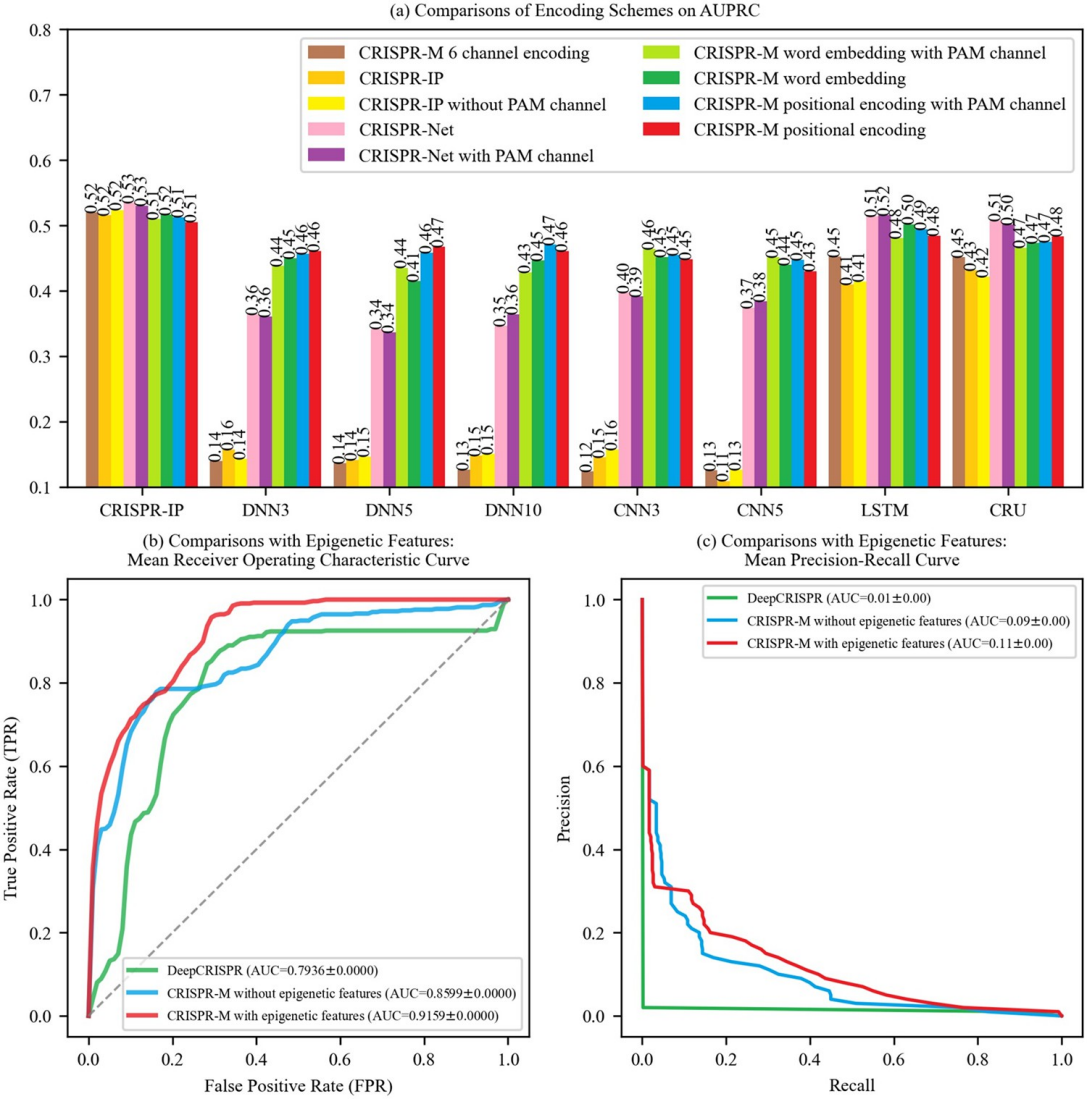
Comparisons of encoding schemes and epigenetic features.

In this section, we test the AUPRC performance of encoding schemes we adopt against the encoding schemes used in CRISPR-Net and CRISRP-IP, using the CIRCLE dataset for LOGOCV. Nine encoding schemes are compared: (a) The six-bit manual encoding scheme extends the compression encoding of CRISPR-Net and CRISRP-IP, depicted as "CRISPR-M 6 channel encoding". In particular, we construct the encoding scheme based on ONE-HOT and the OR operation, by converting the two-bit direction channel into one-bit; (b) the manual encoding scheme for CRISPR-IP, depicted as "CRISPR-IP"; (c) the manual CRISPR-IP encoding without the PAM channel, depicted as "CRISPR-IP without PAM channel". Note that the PAM channel is a one-bit encoding proposed by CRISPR-IP to indicate whether the current base is in the guide sequence region or the PAM sequence region; (d) the manual CRISPR-Net encoding scheme, depicted as "CRISPR-Net"; (e) manual CRISPR-Net encoding added with a one-bit PAM channel, depicted as "CRISPR-Net with PAM channel"; (f) "CRISPR-M word embedding" is the proposed adaptive encoding scheme based on the word embedding; (g) "CRISPR-M word embedding with PAM channel" is an encoding scheme that distinguishes between the guide sequence region and the PAM region in the word embedding dictionary based on encoding scheme (f); (h) "CRISPR-M positional encoding" is an encoding scheme that adds positional encoding to encoding scheme (f); (i) "CRISPR-M positional encoding with PAM channel" is an encoding scheme that adds positional encoding to encoding scheme (g). More details can be found in "Encoding Scheme" of Section “Methods”. As shown in Fig 3(A), DNNx denotes a neural network composed of x fully connected layers, CNNx denotes a neural network composed of x convolutional layers and two fully connected layers, LSTM denotes a neural network composed of one LSTM layer and two fully connected layers, and GRU denotes a neural network composed of one GRU layer and two fully connected layers. We also introduce the CRISPR-IP model in the experimental comparisons since it contains one or two layers for each network module.

Three main issues are evaluated in the experiments: (1) comparisons between a manual encoding scheme and an adaptive encoding scheme based on the word embedding; (2) whether adding a one-bit PAM channel to the encoding scheme is benefit to the network model performance; (3) whether adding positional encoding is a benefit to the network model performance.

For issue (1), as shown in Fig 3(A), for the CRISPR-IP model, the adaptive encoding schemes (f), (g), (h) and (i) outperform the manual encoding schemes (a), (b) and (c). The encoding schemes (f), (g), (h) and (i) outperform manual encoding schemes (a), (b), (c), (d) and (e) for the fully connected models and convolutional models, and the performance difference is even larger than the CRISPR-IP model and the recurrent layer-based models. The CRISPR-IP model and the recurrent layer-based models have more powerful learning capability than the fully connected models and convolutional models, which reduces the performance difference between manual and adaptive encoding schemes. Therefore, we adopt the adaptive encoding scheme based on the word embedding.

For issue (2), four groups of control encoding schemes with and without PAM channels, which are {encoding schemes (b) and (c)}, {encoding schemes (d) and (e)}, {encoding schemes (f) and (g)}, {encoding schemes (h) and (i)}, are evaluated in the experiments. As shown in Fig 3(A), there is no obvious performance difference between two encoding schemes within each control group. At this point, we find that the PAM channel has little influence in network model performance. The reason is that neural networks have the ability of recognizing locations. On the contrary, adding the PAM channel needs more consumption in space and time of training the model. Therefore, we adopt the encoding scheme without the PAM channel for model training.

For issue (3), two groups of control encoding schemes with and without positional encoding, which are {encoding schemes (f) and (h)} and {encoding schemes (g) and (i)}, are evaluated in the experiments. As shown in Fig 3(A), for the first group, the encoding scheme (h) with positional encoding performs worse than that of the encoding scheme (f) in CNN3, but better than (or equal to) that of the encoding scheme (f) in the rest network models. For the second group, the encoding scheme (i) with positional encoding performs obviously outperforms encoding scheme (g) in DNN5, and has similar performance with encoding scheme (g) in the rest network models. Therefore, we adopt the encoding scheme with the positional encoding for model training.

In summary, we adopt the adaptive encoding scheme based on the word embedding and the positional encoding without the PAM channel (i.e., encoding scheme (i)) for model training.

### Comparisons with epigenetic features

In this section, we compare the performance of CRISPR-M with previous representative approach DeepCRISPR, applying epigenetic features (CTCF, DNase, H3K4me3 and RRBS [27]) and sequence features to predict sgRNA off-target effect. We test the performance of DeepCRISPR, CRISPR-M with sequence features only (depicted as CRISPR-M without epigenetic features), and CRISPR-M with sequence and epigenetic features (depicted as CRISPR-M with epigenetic features), using dataset K562 [27] and dataset HEK293T [27]. As shown in Fig 3B–3E, CRISPR-M with epigenetic features shows better performance than CRISPR-M without epigenetic features, and both of them outperforms DeepCRISPR, in terms of ROC and PRC. This further demonstrates that CRISPR-M has good extensibility and adding epigenetic features could improve sgRNA off-target effect predictions.

### Impact of random seed on AUPRC results and ablation experiments

**Fig 4:**
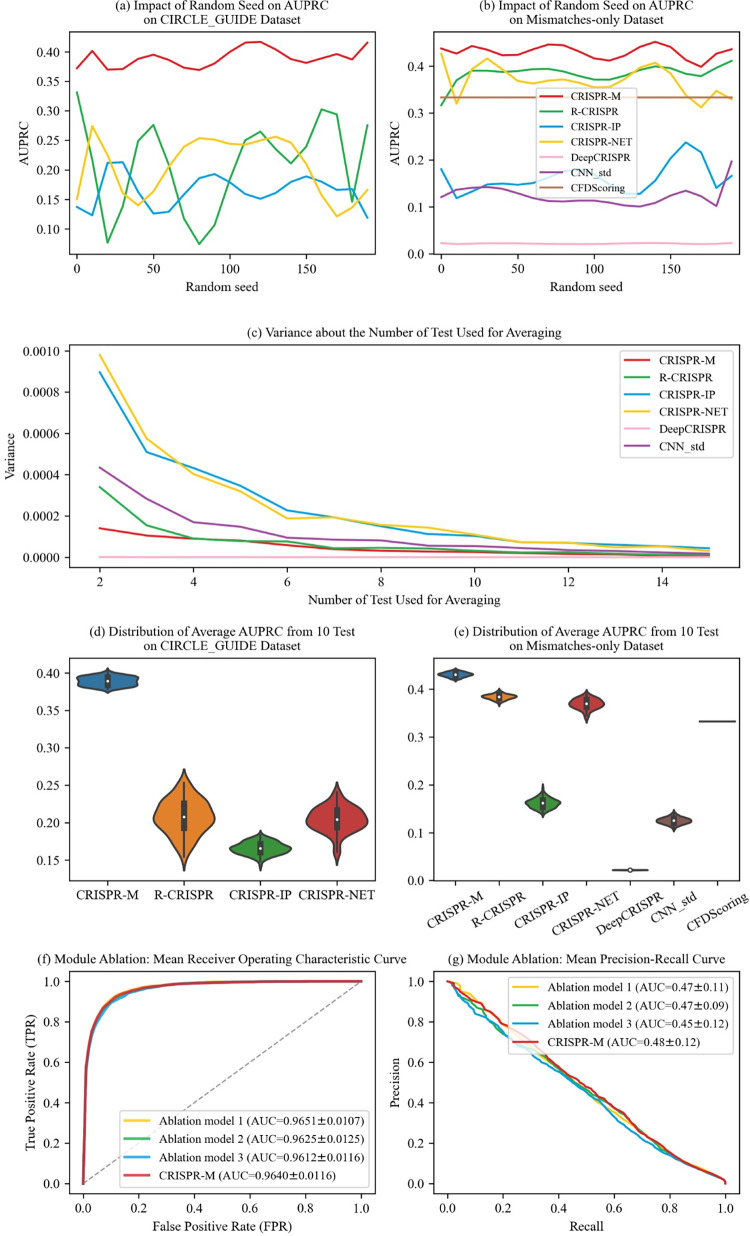
Impact of random seed on AUPRC results, and results of ablation experiments.

In this section, we examine the influence of random seed selection on the results of the AUPRC. In Fig 4(A) and 4(B), we assess the AUPRC results based on different random seeds, running on the CIRCLE_GUIDE dataset and the Mismatches-only dataset. The horizontal axis of Fig 4(A) and 4(B) are random seed values, and the vertical axis represents the corresponding AUPRC values. We observe that the AUPRC results of CRISPR-M are generally better than other methods when using different random seeds. Fig 4(C) shows the variance curves of average AUPRC results, where the horizontal axis is the number of AUPRC results used for calculating the averages, and the vertical axis is the corresponding variance of the averages. We observe the variance curve becomes stable at 10 trials. Since the results are qualitatively similar after 10 trials, for simplicity, we also choose the average of 10 trial results for comparisons in previous experimental sections. Fig 4(D) and 4(E) illustrate the distribution of average AUPRC values of tested methods. We observe that the results of tested methods are stabilized within a narrow range, and CRISPR-M still performs better than other approaches, which is consistent with the experimental results in previous sections.

In the following, we perform ablation experiments to demonstrate the effects of each module. In particular, by removing three parts of the CRISPR-M model (i.e., the CNN module, the RNN module and the fully connected module), we build three ablation models: "Ablation model 1", "Ablation model 2", and "Ablation model 3", where "Ablation model 3" retains a dense layer as the output layer. The experimental results are shown in Fig 4(F) and 4(G). The AUROC values show minor differences among the models, and the ablation models exhibit a decreased performance in AUPRC values compared with CRISPR-M, demonstrating the effectiveness of each module in CRISPR-M.

### Visual analysis of CRISPR-M on the off-target effect prediction

In this section, we adopt CRISPR-M trained with datasets CIRCLE and SITE to visually analyze the influence of number and position of mismatches and indels, GC content and melting temperature, in terms of sgRNA off-target effect. To visualize the influence of the factors above, we randomly generate 10,000 on-target sequences and set PAM sequences to "AGG" for simplicity. For each on-target sequence, we replace one of the twenty-three base sites at a time with another three bases or an indel, constructing 92 off-target sequences associated with the on-target sequence.

**Fig 5:**
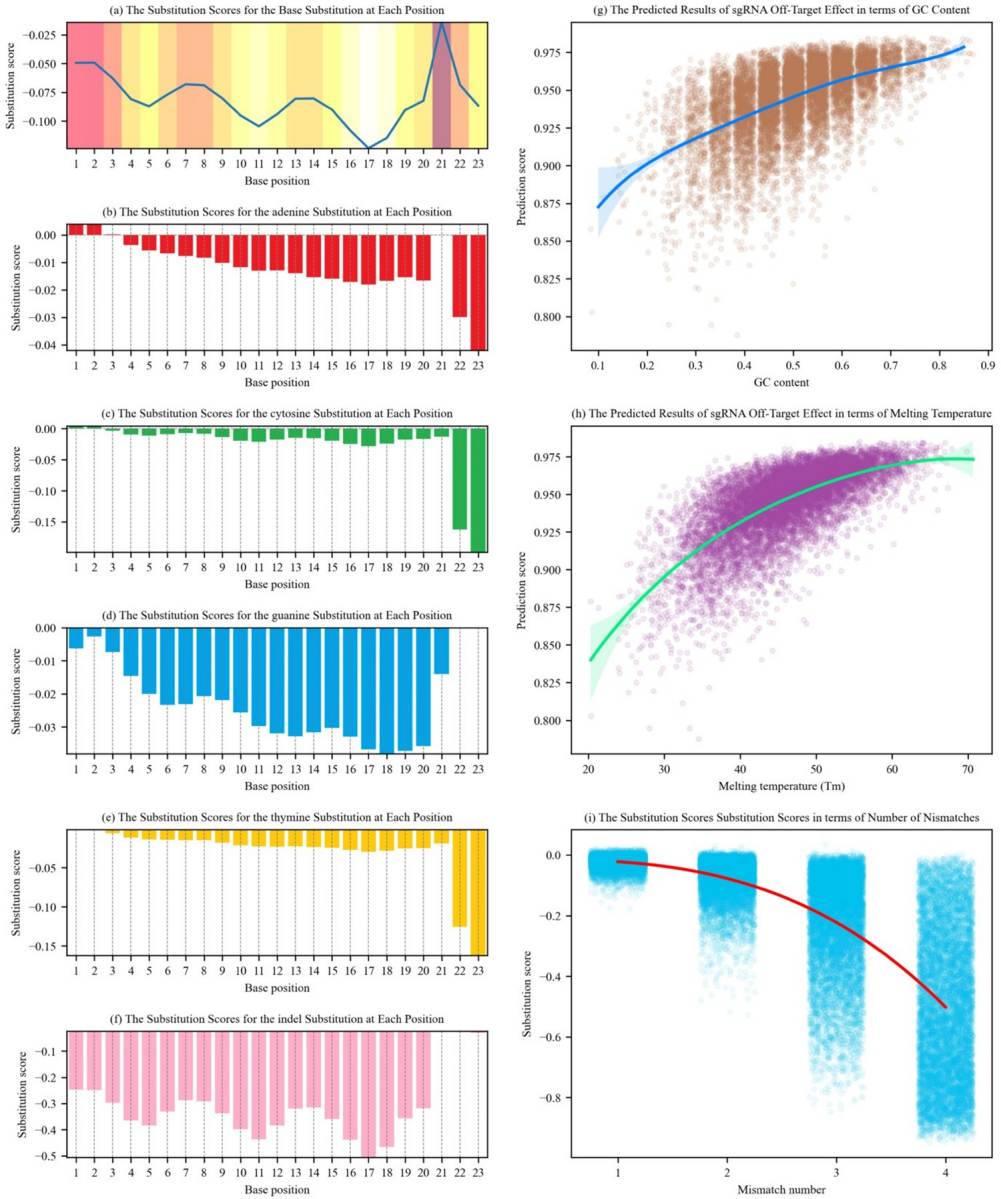
Visual analysis of CRISPR-M. (a) The average substitution scores for the base substitution at each location. (b-f) The substitution scores for the substitution regarding A, C, G, T and an indel. (g-i) The dot plots of the average substitution scores associated with the GC content, melting temperature and mismatch numbers.

We use CRISPR-M to output the difference of predicted values (the predicted value of pair of on- and off-target sequences minus that of on- and on-target sequences), depicted as substitution score, which is used to represent the influence of different base mismatches or an indel on the off-target effect. Fig 5A–5F show the average substitution scores of the 10,000 generated on-target sequences with mismatches or indels at different target sequence positions, where the horizontal axis represents the positions of the target sites and the vertical axis records the average substitution scores (lower score means less possibility of off-target). Fig 5G–5I show the dot plots of the average substitution scores associated with the GC content, melting temperature and mismatch numbers, respectively.

In Fig 5(A), we observe three valleys from 1 to 7, from 8 to 13, and from 14 to 20 in horizontal axis. The closer these three valleys approach to the PAM region, the deeper they are (i.e., the less possibility of off-target). These results are consistent with previous studies. In particular, previous studies [36] illustrate that mismatches in seven to nine positions near the PAM region could result in the less possibility of off-target at a target site. In addition, previous studies [37,38,39,40] illustrate that mismatches in ten to thirteen positions near the PAM region are determinants of the specificity of CRISPR cleavage. Moreover, previous studies [41,42,43] illustrate that the seven positions distal to the PAM region have a low effect on the off-target effect. These results further validate that CRISPR-M could effectively capture features.

Fig 5B–5F show substitution scores caused by a base mismatch or an indel at target sequence positions. In Fig 5(C) and 5(E), the substitution scores decrease greatly when the mismatch occurs at the second position of the PAM region (see horizontal axis 22 in the figures, the corresponding mismatch base pairs are "GC" and "GT" respectively), because a CRISPR system using "NGG" as the PAM sequence has a low possibility of off-target at the off-target site. As shown in Fig 5(B), when the mismatch base pair occurring at horizontal axis 22 is "GA", the substitution score decreases slightly, meaning that the off-target site could be still active. Because "NAG" can be also used as a PAM sequence, CRISPR-M is more tolerant of "GA" than that of "GC" and "GT" at horizontal axis 22. These results are consistent with the previous study [44]. The substitution scores at horizontal axis 22 and 23 in Fig 5(B) are higher than those in Fig 5(C) and 5(E), and the tolerance of the PAM region to adenine has been demonstrated in previous study of CRISPR systems [45]. In Fig 5(F), we observe that the CRISPR system is very intolerant of indels, i.e., there is a small possibility of activating off-target sites containing indels.

Fig 5(G) shows the predicted results of sgRNA off-target effect in terms of GC content using CRISPR-M, where higher predicted values indicate more off-targeting possibility. We observe that higher GC content results in more stable hybridisation of RNA and DNA, and lower GC content leads to less off-target possibility. The fitted curve in Fig 5(G) shows that the predicted values of sgRNA off-target effect increase, with the increases of GC content. This is consistent with previous findings [46,47]. Fig 5(H) shows the predicted results of sgRNA off-target effect in terms of melting temperature between the sgRNA and the off-target site using CRISPR-M, where higher predicted values also indicate more off-targeting possibility. The fitted curve in Fig 5(H) illustrates that the predicted values of sgRNA off-target effect increase, with the increases of melting temperature. This is also consistent with previous findings [20,34,35,48,49]. Fig 5(I) shows the substitution scores substitution scores in terms of number of mismatches using CRISPR-M. The fitted curve in Fig 5(I) shows that the substitution scores decrease, with the increases of number of mismatches, which is consistent with the previous findings [11].

In summary, the visualization results above validate that CRISPR-M could effectively capture features, and these results are consistent with the expected properties that a CRISPR system should have, and also validate existing findings derived from previous gene editing studies.

## Discussion

In this paper, we present CRISPR-M, a multi-view deep learning approach to predict the sgRNA off-target effects for target sites containing indels and mismatches. Our main contributions are as follows. We firstly propose a multi-view learning strategy for the prediction of sgRNA off-target effects, i.e., encoding on- and off-target sequence pairs, on-target sequences and off-target sequences as three input features for model training. Then, we propose an adaptive encoding scheme based on the word embedding and the positional encoding. Next, we propose a multi-branch deep learning model based on multiple network structures towards the multi-view strategy and adaptive encoding scheme. Experimental results demonstrate that CRISPR-M outperforms previous sgRNA off-target effect prediction approaches, and has good generalization capability, when handling mismatches and indels. In addition, we perform comparisons from perspectives of encoding scheme and epigenetic features. CRISPR-M shows the effectiveness and advantages of its encoding scheme, and achieves promising results in handling both sequence and epigenetic features. Finally, we perform a visual analysis of CRISPR-M to verify its validity by evaluating the influence of number and position of mismatches and indels, GC content and melting temperature, in terms of sgRNA off-target effect.

## Methods

**Fig 6:**
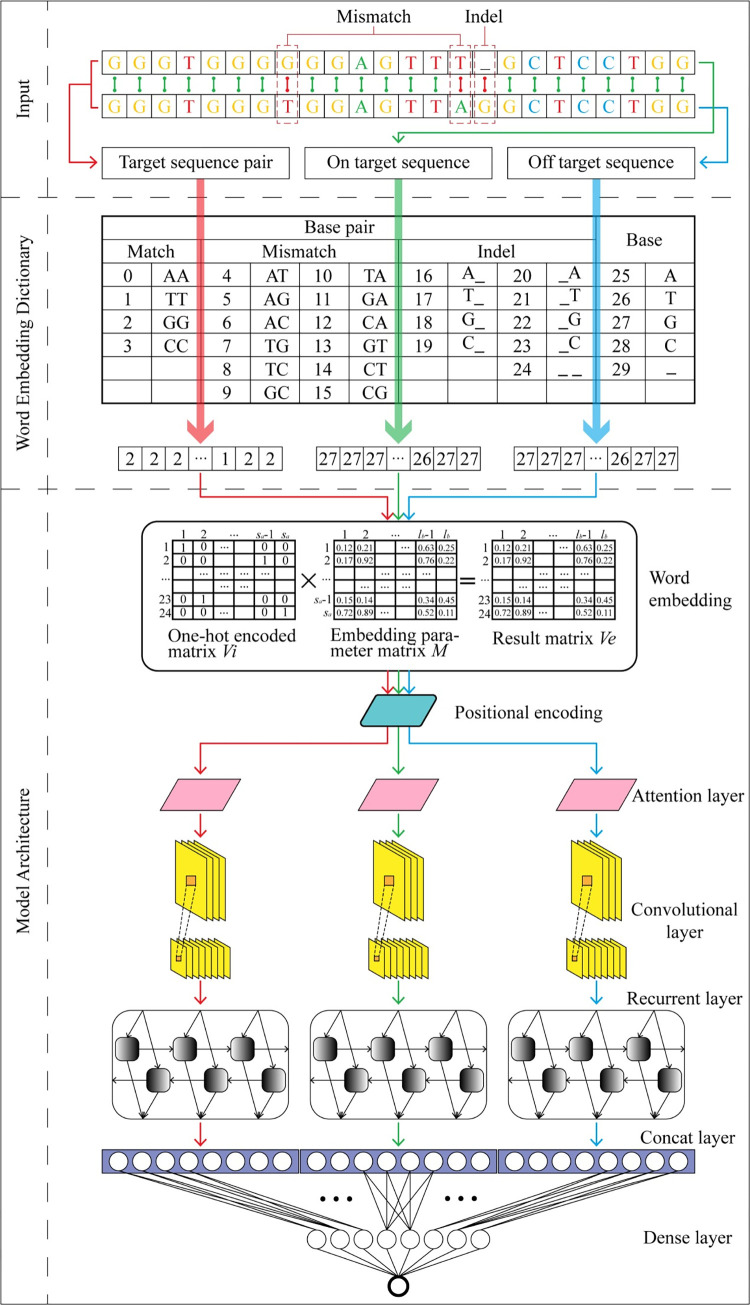
Overview of CRISPR-M.

CRISPR-M contains convolutional layers, recurrent layers, attention layers, fully connected layers, regularization strategies and the word embedding and positional encoding layers. The overview structure of CRISRP-M is shown in Fig 6.

### Multi-view learning and encoding schemes

As shown in the Input part of Fig 6, we encode the on- and off-target sequence pairs, the on-target sequences, and the off-target sequences as three input features, representing three perspectives: the association between the on- and off-target sequences, the features of the on-target sequences, and the features of the off-target sequences. We integrate these features above and form a multi-view learning scheme regarding sgRNA off-target effect predictions.

In the following, we introduce the adaptive encoding scheme of CRISPR-M, based on word embedding and positional encoding.

Compared with the one-hot encoding scheme, the word embedding encoding allows for the encoding of discrete information in a distributed representation way and could adaptively adjusts the distance between discrete information, such as the distance among the four bases in the Euclidean space. Specifically, we design a dictionary, as shown in the Word Embedding Dictionary module in Fig 6, for converting bases and base pairs into the word indexes for the word embedding. To accommodate the indel information in our encoding scheme, another 1-nt beside the 20-nt target sequence and the 3-nt PAM sequence is added to form a 24-nt base sequence or base pair sequence. As shown in Fig 6, a 24-nt base pair sequence is entered into the embedding layer, after it has been encoded as a word index vector. The encoding formula in the embedding layer is as follows.


$$V_{e}=V_{i}\times M$$ (9)

Here, *V*_*i*_ (“One-hot encoded matrix” in Fig 6) denotes the vector of word indexes encoded by using the one-hot encoding associated with the serial number in the dictionary. Assume that the size of the dictionary is *s*_*a*_, the shape of *Vi* is [24, *s*_*a*_]. Suppose that the word embedding length is *l*_*b*_, the shape of the embedding layer parameter matrix M (“Embedding parameter matrix” in Fig 6) is [*s*_*a*_, *l*_*b*_], and the shape of the word vector matrix *V*_*e*_ (output from the embedding layer) is [24, *l*_*b*_]. In addition, since different positions of target sites have different influence on off-target effects, we have included positional encoding. The formula used for the positional encoding is as follows:

$$PE_{\left(pos,i\right)}=\left\{ \begin{array}{cc} \sin\left(pos/10000^{i/d}\right), & i\;mod\;2=0\\ \cos\left(pos/10000^{i/d}\right), & i\;mod\;2=1 \end{array}\right.$$ (10)

Here, *pos* denotes the position of the base or base pair in the target sequence, *i* denotes the dimension in the base or base pair word vector, and *d* denotes the maximum dimension of the word vector. For a 24-nt sequence with a word vector length of *l*_*b*_, there are 24 *pos* values, and there are *l*_*b*_ values for *i* and d is equal to word vector length *l*_*b*_. Formula 10 gives each value in the word vector matrix *V*_*e*_ a unique position. The entire encoding process is completed by inputting the word vector matrix *V*_*e*_ into the positional encoding layer and outputting it after adding the positional encoding. The corresponding encoding formula is as follows.


$$V=V_{e}+V_{p}$$ (11)

Here, *V*_*p*_ is the position matrix of the same shape as *V*_*e*_, calculated from Formula 10. *V* is the matrix of the final encoding output. So far, we obtain an encoding containing location information that can be adaptive to the distance between discrete information.

### Model architecture

Firstly, the word embedding dictionary encodes the on- and off-target sequence pair, the on-target sequence and the off-target sequence as three inputs to the embedding module. These three inputs are encoded in the word embedding and positional encoding layers. Subsequently, each input associate with a branch. At the beginning of each branch, a self-attention layer is used to reinforce the features of each input, facilitating feature extractions of subsequent convolutional layers. The self-attention layer uses multi-head attention as follows.


$$Attention(Q,K,V)=softmax\left(\frac{QK^{T}}{\sqrt{d_{k}}}\right)V$$ (12)

$$head_{i}=Attention(QW_{i}^{Q},KW_{i}^{K},VW_{i}^{V})$$ (13)

$$MultiHeadAttention(Q,K,V)=Concat(head_{1},\ldots ,head_{h})W^{O}$$ (14)

*Q*, *K* and *V* denote the queries and keys in the dimension *d*_*k*_ and the values in the dimension *d*_*v*_, respectively. *K*^*T*^ is the transpose matrix of *K*. Softmax function is used to transform matrix product into probability. $W_{i}^{Q},\;W_{i}^{K},\;W_{i}^{V}$ and *W*^*O*^ in Formula 13 represent the parameter matrix corresponding to *Q*, *K*, *V* and matrix generated from concatenated heads. Concat function is used to concatenate the matrixes corresponding to multiple heads. Formula 14 represents a multi-head attention consisting of multiple weighted attentions.

Next, we design convolutional layers behind the attention layers for feature extractions. The number of filters per convolutional layer is set to 32 or 64. For each sample, the size of the vector output from the attention layer is [24, *l*_*b*_]. We reshape the output of the attention layer before the convolution layer as [24, *l*_*b*_, 1]. In each branch, one or two convolutional layers output a tensor of shape [24, 1, *f*_*n*_] (*f*_*n*_ denotes the number of filters) after extracting the features. This tensor is reshaped into [24, *f*_*n*_] and fed into a bi-directional recurrent layer with a cell number which is equal to 32. As shown in Fig 6, the outputs of the recurrent layers of three branches are flattened and concatenated together, resulting in a single prediction output through three fully-connected layers whose cell numbers are 256, 64 and 1 respectively. The detailed model architecture and parameters can be viewed in our GitHub repository.

In the training process, we set Adam [50] as the optimizer, Accuracy, AUROC (Area under ROC curve) and AURPC (Area under PRC) as the model evaluation metrics, and the binary cross-entropy as the loss function. The corresponding formula is as follows:

$$BCE=-\frac{1}{n}\sum_{i}^{n}y_{i}\times log\;{\hat{y}}_{i}+(1-y_{i})\times log\;(1-{\hat{y}}_{i})$$ (15)

Here, *n* denotes the length of the output result, *i* is each bit of the output value, and *y*_*i*_ and *ŷ*_*i*_ denote the real and predicted values respectively.
